# Detection of *Promyelocytic Leukemia/Retinoic Acid Receptor α* (*PML/RARα*) Fusion Gene with Functionalized Graphene Oxide

**DOI:** 10.3390/ijms140612863

**Published:** 2013-06-20

**Authors:** Ran Li, Yanhong Tan, Xiuhua Chen, Fanggang Ren, Yaofang Zhang, Zhifang Xu, Hongwei Wang

**Affiliations:** Department of Hematology, the Second Hospital of Shanxi Medical University, Taiyuan 030001, China; E-Mails: rli@sxicc.ac.cn (R.L.); tanyh1979@hotmail.com (Y.T.); chenxiuhua1996@sohu.com (X.C.); fanggang098@163.com (F.R.); zhyf125@163.com (Y.Z.); xu_zfang@163.com (Z.X.)

**Keywords:** *PML*, *RARα* fusion gene, graphene oxide, fluorescein isothiocyanate (FITC), laser confocal microscopy

## Abstract

An attempt was made to use functionalized graphene oxide (GO) to detect the *Promyelocytic leukemia/Retinoic acid receptor α* fusion gene (*PML/RARα* fusion gene), a marker gene of acute promyelocytic leukemia. The functionalized GO was prepared by chemical exfoliation method, followed by a polyethylene glycol grafting. It is found that the functionalized GO can selectively adsorb the fluorescein isothiocyanate (FITC)-labeled single-stranded DNA probe and quench its fluorescence. The probe can be displaced by the *PML/RARα* fusion gene to restore the fluorescence, which can be detected by laser confocal microscopy and flow cytometry. These can be used to detect the presence of the *PML/RARα* fusion gene. This detection method is verified to be fast, simple and reliable.

## 1. Introduction

Nanotechnology is a cutting-edge high technology of the 21st century, which is being used more and more in the field of medical diagnostics [1]. Graphene is the world’s thinnest novel two-dimensional nanomaterials with a thickness of only 0.35 nm. It becomes a hotspot in the field of nanotechnology and has many important potential applications in composite materials field [2–4], sensors and energy, *etc.* [5–8], owing to its excellent electrical [9,10], mechanical and thermal properties [11–13]. Geim and Novoselov [14], the discoverers of graphene, won the 2010 Nobel Prize in Physics. The research of graphene in the biomedical application began in the recent years. The graphene oxide (GO), an oxidative derivative of graphene, has oxygen-containing functional groups in graphene edge or plane, such as hydroxyl, carbonyl, carboxyl [15,16]. The presence of these functional groups in GO makes it biocompatible, stable in hydrophilic solution, and compatible with polymers. These groups are also conducive to the chemical functionalization to achieve targeted applications in different areas, especially in biomedical field [17–28].

At present, many scholars have studied on the application of the GO in drug loading systems, biological detection, bio-imaging and cancer treatment as well as its biological safety. In the biological detection, the GO can be used to detect DNA, ions, small molecules and proteins, *etc.* [1]. GO is a carrier and has the characteristics of quenching fluorophores [29] in detection.

Acute Promyelocytic Leukemia (APL) is an acute leukemia, which is often accompanied by severe bleeding. More than 95% APL results from the specific chromosomal translocation t (15; 17) (q22; q12~21) so that the *promyelocytic leukemia* (*PML*) gene on the 15th chromosome and the *retinoic acid receptor α* (*RARα*) gene on the 17th chromosome fuse together to form the *PML/RARα* fusion gene, which is a marker of APL in the early diagnosis and prognostic monitoring [30]. The detection methods of the *PML/RARα* fusion gene include chromosome analysis [31], real-time quantitative RT-PCR [32], FISH, *etc*. However, low specificity, complicated operation and the high cost of these testing methods limit their wide applications.

**Figure 1:**
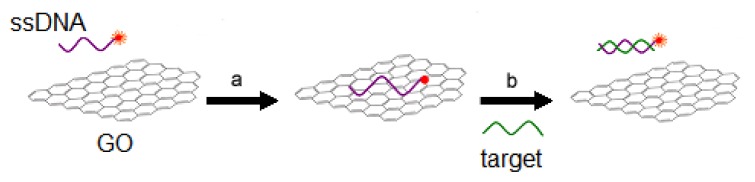
Schematic representation of the target-induced fluorescence change of the single-stranded DNA (ssDNA)–graphene oxide (GO) complex.

GO has a high adsorption capacity for the probe with fluoresce-labeled single-stranded DNA (ssDNA), and can quench its fluorescence (step **a** in Figure 1). In the presence of a target molecule, the ssDNA labeled by the fluorescent, can be detached from the GO, and the fluorescence of the probe is restored (step **b** in Figure 1) [33]. So GO can be used to the detection of the *PML/RARα* fusion gene by measuring fluorescence intensity.

The purpose of this paper is to determine how specific the GO is in the detection of the *PML/RARα* fusion gene.

## 2. Results and Discussion

### 2.1. Preparation of the Functionalized GO and Its Stability in Biological Solutions

As-synthesized GO with reference to the method of Zhang *et al.* [34] has a thickness of about 1–2 nm and a width of about 100 nm. The dispersion ability of the GO is different from the functionalized GO in the water, PBS solution, cell culture medium and the serum. The dispersion of the GO is good in aqueous solution, but the GO is easy to agglomerate in the solution rich in salt and protein, such as cell culture medium and serum. However, the functionalized GO is stable in these biological solutions, owing to the non-specific binding of charge and proteins. So, the functionalized GO, (GO–PEG) is used in our experiments.

### 2.2. The Single-Stranded DNA Fluorescence Quenched by the Functionalized GO and Recovered by the Target Molecule *in Vitro* without Cells

**Figure 2:**
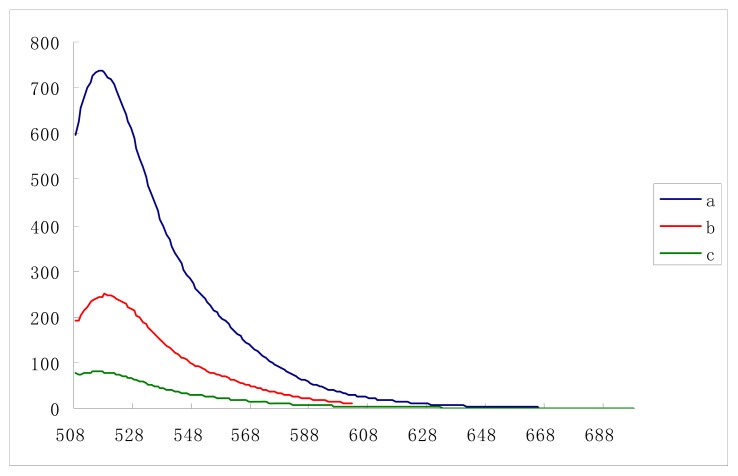
Fluorescence emission spectra of probe in the (**a**) absence, (**c**) presence of GO and (**b**) after target molecule was added to the fluorescence quenched solution by GO. The concentrations of probe were 50 nM. The concentrations of target molecule were 100 nM.

The GO has strong affinity with the fluorescence-labeled single-stranded DNA probe and can quench its fluorescence. After GO reacts with the target molecule, the DNA labeled by the fluorescent probe, can be detached from the GO, and the fluorescence of the probe is restored as shown in Figure 2.

### 2.3. The Single-Stranded DNA Fluorescence Quenched by the Functionalized GO and Recovered by the Target Molecule with Cells

**Figure 3:**
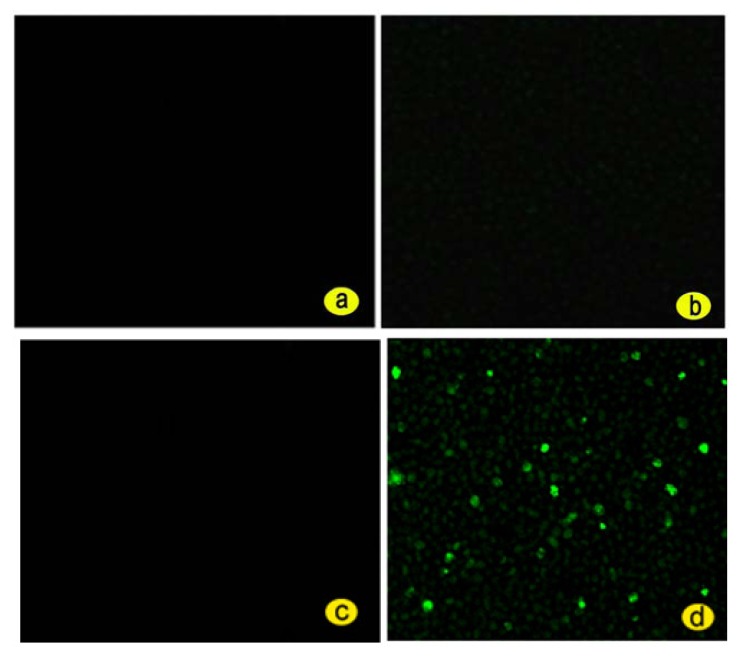
The laser confocal microscopic images of the control K562 group cells (**a**) without and (**b**) with Intervention of the fluorescein isothiocyanate (FITC)-labeled single-stranded DNA probe, and the images of the experimental NB4 group cells (**c**) without and (**d**) with Intervention of the FITC-labeled single-stranded DNA probe.

**Figure 4:**
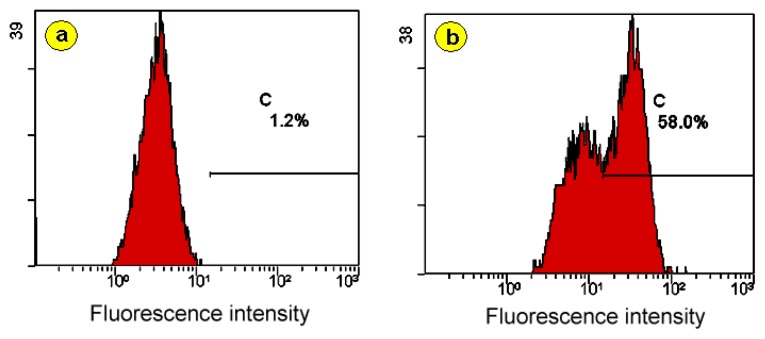
The flow cytometric results of the functionalized GO adsorbed experimental NB4 group cells with the *PML/RARα* fusion gene (**a**) without and (**b**) with intervention of the FITC-labeled single-stranded DNA probe.

In order to verify how specific the functionalized GO is for the detection of the *PML/RARα* fusion gene, the experimental NB4 group cells and the control K562 group cells were all divided into the negative group with no intervention with fluorescein isothiocyanate (FITC)-labeled single-stranded DNA and the positive group intervened by FITC-labeled single-stranded DNA as shown below in Table 1. All cell groups together with the incubation medium in Table 1 were analyzed by a laser confocal microscopy and a flow cytometry after incubation. The results are shown in Figures 3 and 4.

#### 2.3.1. The Results with Laser Confocal Microscopy and the Analysis of Results

It is found that there are no green fluorescence in Figure 3a,c and there is scattered green fluorescence in Figure 3d, indicating that there are cells of the *PML/RARα* fusion gene in the experimental NB4 group cells. Figure 3a is the image of the control K562 group cells without intervention of the FITC-labeled single-stranded DNA probe, so there is no fluorescence signal. In Figure 3b, there is very poor fluorescence signal, because there is no *PML/RARα* fusion gene in K562 Group Cells, However when fluoresce-labeled single-stranded DNA encounter noncomplementary sequence, there was a significantly low fluorescence signal as a background signal [33]. Although there is *PML/RARα* fusion gene in the experimental NB4 group cells, there is no green fluorescent signal as shown in Figure 3c since the FITC-labeled single-stranded DNA probe was not intervened. And there is green fluorescent signal in the experimental NB4 group cells as shown in Figure 3d if the FITC-labeled single-stranded DNA probe was intervened since the *PML/RARα* fusion gene combines with the FITC-labeled single-stranded DNA probe quenched by the functionalized GO and the fluorescence emission of the probe is restored.

#### 2.3.2. The Results with Flow Cytometry and the Analysis of Results

Figure 4a,b show the flow cytometric results of the negative and positive NB4 group cells without and with intervention of the FITC-labeled single-stranded DNA probe respectively. It is found that when the FITC-labeled single-stranded DNA probe is intervened the number of FITC-positive cells is 56.00% ± 8.19% and the number is 1.20% ± 0.20% when the probe is not intervened, indicating that the difference between the number of the FITC-positive cells for the positive and negative groups is statistically significant (*F* = 134.384, *p* = 0.000), as shown below in Table 2.

The possible reasons why not all of the *PML/RARα* fusion genes were detected in the positive group are as follows. Firstly, part of the fusion cells was lost by Saponin hormone during pretreatment of the cells. Second, part of the functionalized GO carrying the probe could be lost during washing centrifuging with PBS buffer. Lastly, a few of the fluorescent probes were not released by competitive adsorption with the fusion gene in the functionalized GO. Further experiments are needed to verify these potential explanations.

Nevertheless, the detection of the *PML/RARα* fusion gene with the functionalized GO is reliable, manifested by the statistical significant between the positive and negative as well as experimental and control group cells.

At present, the detection techniques of the *PML/RARα* fusion gene have conventional chromosome analysis, RT-PCR and FISH analysis. Conventional chromosome analysis for t (15;17) has disadvantages such as complexity of specimen preparation and less observed mitosis. Although RT-PCR technique has a high sensitivity, many influence factors lead to a high false negative and positive rate [35]. FISH analysis has a strong specificity [36], but the operation is complex, time consuming, and also it is expensive. Compared with these traditional methods, the detection of our experiment not only has a high specificity, but also the operation is very simple, cost is very low and the time required for analysis is short, only about 1.5 h is enough to get the reliable result. These findings encourage us to carry out future work to use the functionalized GO in the field of molecular biology and medical diagnostics.

## 3. Materials and Methods

### 3.1. Preparation of GO

GO was prepared according to the chemical exfoliation method. One gram of graphite powder was put into a beaker, to which 0.75 g NaNO_3_ and 75 mL concentrated H_2_SO_4_ were added. The beaker was placed into an ice bath and the mixture in the beaker was added slowly with 5 g KMnO_4_ with stirring within 1 h, followed by a stirring for 2 h in the ice bath and 4 days at room temperature. The beaker was again put into the ice bath and 2.2 g KMnO_4_ was added slowly with a stirring to the beaker. The stirring continued for 30 min after the addition.

The temperature of the bath was risen to 98 °C and 140 mL 5% diluted H_2_SO_4_ was slowly added with stirring into the beaker within 1 h with a consecutive stirring for 2 h after the addition. The temperature of the bath was lowered to 60 °C and 100 mL 30% H_2_O_2_ was added with a stirring to the beaker. The stirring continued for 2 h at room temperature after the addition.

The resulting mixture was centrifuged for 10 min at 4000 rpm at room temperature. The supernatant was discarded and 480 mL 3% dilute HCl was added into the precipitate to form a mixture that was centrifuged for 8 min at 4000 rpm at room temperature for twice. The supernatant was discarded and the precipitate was repeatedly washed with the sterilized distilled water until the pH value of the supernatant turned to neutral. The precipitate collected was put into a beaker, to which 120 mL of the sterilized distilled water was added to form a suspension that was ultrasonicated for 1 h with an ultrasonic probe of a cell disrupter. The suspension was centrifuged and the supernatant obtained was a GO solution.

### 3.2. Functionalization of GO with Polyethylene Glycol

Five milliliter of the above GO solution was added with 3 M NaOH and ultrasonicated for 4 h. The solution was neutralized by the HCl until the pH value turned to neutral. The solution was added with polyethylene glycol (PEG) to a concentration of 10 mg/mL and ultrasonicated for 10 min. The solution was first added with *N*-Ethyl-*N*,-(3-dimethylaminopropyl)-carbodiimide, hydrochlorid (EDAC HCl) to a concentration of 5 mmol/L and ultrasonicated for 30 min, then to a concentration of 20 mmol/L and stirred for 12 h. Finally, mercaptoethanol was added into the solution to terminate the reaction. The resulting solution is a PEG functionalized GO solution.

### 3.3. Design of Probe

A fluorescence-labeled single-stranded DNA probe, 5′-FITC-TGACCTGCCATTGAG-3′, is designed according to *PML/RARa* fusion gene fracture fusion point in our experiments. The fluorescence-labeled single-stranded DNA probe was synthesized by Shanghai Sangon Biotechnology Co.

### 3.4. Fluorescence Quenching and Hybridization Assays

The working probe solution containing the fluorescence-labeled single-stranded DNA was diluted to a concentration of 50 nM by using Tris-HCl buffer (20 mM, pH 7.4, containing 100 mM NaCl, 5 mM KCl, 5 mM MgCl_2_) [37]. The GO solution (about 0.04 mg/mL) was added to the working probe solution and incubated for 5 min. Exactly100 nM of the target molecule was then added to the GO–DNA mixture and allowed to hybridize for 1 h at room temperature. The fluorescence spectra of the mixed solutions with or without GO and before or after addition of target molecule were detected with a CARY Elipse VAPIAN flouresence spectrophotometer.

### 3.5. Cell Culture

The experimental NB4 group cells and the control K562 group cells were seeded in RPMI-1640 culture medium containing 10% fetal bovine serum and cultured at 37 °C with 5% CO_2_ with half of the culture medium exchanged after 2–3 days. Before the experimental and control cells were counted the density of the cells was adjusted according to the experimental requirements.

### 3.6. Cell Pretreatment

The experimental NB4 cells and the control K562 cells were separately collected into two centrifugal tubes. The number of the cells in each centrifugal tube was approximately 2 × 10^6^. The tubes were centrifuged for 5 min at 1000 rpm at room temperature, and the supernatant was discarded. Each tube was added with 100 μL formaldehyde and mixed immediately. After the mixture was allowed to stand for 10–15 min at room temperature, the same volume of PBS buffer was added and centrifuged to discard the supernatant. Then each tube was added with 100 μL Saponin hormone. After the mixture was allowed to stand for 5 min at room temperature, the same volume of PBS buffer was again added gently and centrifuged. Finally, the supernatant was discarded and the mixture was kept on standby.

### 3.7. The Incubation Medium and the Cells Were Incubated

The liquids listed in Table 1 were added to the above-treated cells, thoroughly mixed, and allowed to stand for 1 h at room temperature in the condition of darkness. The mixture was centrifuged for 5 min at 1000 rpm, and the supernatant was discarded. After the PBS buffer was added into each centrifuge tube and mixed, the mixture was centrifuged and the supernatant was discarded. Then 1ml PBS buffer was added into each centrifuge tube. After mixing, the mixture was moved to the 24-well plate with a pipette for further detection.

## 4. Conclusions

The present experiment shows an effective method for the detection of *PML/RARα* fusion gene with an aid of the functionalized GO. The functionalized GO is stable in biological solutions and is capable to selectively adsorb and quench the fluorescence of FITC-labeled single-stranded DNA probe. The adsorbed FITC-labeled single-stranded DNA probe can be released by a competitive adsorption of *PML/RARα* fusion gene in NB4 cell, and the fluorescence that can be detected by a laser confocal microscopy and a flow cytometry. These can be used to detect the presence of the *PML/RARα* fusion gene.

## Figures and Tables

**Table 1:** The Formulations of the incubated liquid with Cells.

Reagent	NB4 (negative group)	NB4 (positive group)	K562 (negative group)	K562 (positive group)
GO	0.04 mg/mL	0.04 mg/mL	0.04 mg/mL	0.04 mg/mL
Probe	-	200 nM	-	200 nM
Tris-Hcl buffer	20 mM	20 mM	20 mM	20 mM

Note: The concentration of the Tris-HCl buffer was 20 mM, pH was 7.4, containing NaCl of 100 mM, KCl of 5 mM, and MgCl_2_ of 5 mM.

**Table 2:** The flow cytometry results of the positive and negative experimental NB4 group cells with the *PML/RARα* fusion gene with and without intervention of the FITC-labeled single-stranded DNA probe.

Packet	Interventions			*PML/RARα* fusion gene positive cells (%)	F	P
The negative group	Tris-Hcl buffer	The functionalized GO	-	1.20 ± 0.20	134.384	0.000
The positive group	Tris-Hcl buffer	The functionalized GO	The FITC-labeled single-stranded DNA	56.00 ± 8.19		
